# Supplementary material for: A modular circuit coordinates the diversification of courtship strategies
Source: Nature. 2024 Oct 9;635(8037):142–50. doi: 10.1038/s41586-024-08028-1 (PMC11540906; doi:10.1038/s41586-024-08028-1)
Supplement: Supplementary file 1 — This file contains tables with the experimental genotypes of all males (Supplementary Table 1) and all females (Supplementary Table 2) by figure. [file 41586_2024_8028_MOESM1_ESM.pdf]

nature portfolio

https://doi.org/10.1038/s41586-024-08028-1

# Supplementary information

# A modular circuit coordinates the diversification of courtship strategies

In the format provided by the authors and unedited

Supplementary Table 1. Male experimental genotypes by figure.

| Figure | Species | Male Genotype |
|---|---|---|
| 1b-d; 3d,e,f, Ext1a-d; Ext3c,d; Ext4b; Ext8a,c,d,f,g | *D. yakuba* | Wildtype (Ivory Coast) |
| 1b,c; 3c; Ext1a,c; Ext3b; Ext8a | *D. melanogaster* | Wildtype (Canton S) |
| 1b,c; Ext1a-c | *D. simulans* | Wildtype |
| 1b,c; Ext1a | *D. erecta* | Wildtype (14021-0224.01) |
| 1b,c; Ext1a | *D. eugracilis* | Wildtype (SHL12) |
| 1b,c; Ext1a | *D. ananassae* | Wildtype (14024-0371.34) |
| 2a | *D. melanogaster* | w; 71G01-Gal4/UAS-CD8::GFP |
| 2b | *D. melanogaster* | w; UAS-GCaMP6s/+; 71G01-Gal4/+ |
| 2a, Ext2a | *D. erecta* | UAS-CD8::GFP/+; 71G01-Gal4/+ |
| 2b | *D. erecta* | UAS-GCaMP6s/+; 71G01-Gal4/+ |
| 2a,b | *D. simulans* | w; UAS-GCaMP6s/+; 71G01-Gal4/+ |
| 2a, Ext2b | *D. yakuba* | w; 71G01-AD/+; 15A01-DBD/UAS-CD8::GFP |
| 2b | *D. yakuba* | w; 71G01-AD/UAS-GCaMP6s; 15A01-DBD/+ |
| 3a; Ext3e | *D. yakuba* | w; ppk23-Gal4/UAS-CD8::GFP |
| 3a; Ext3e | *D. melanogaster* | w; ppk23-Gal4/+; UAS-CD8::GFP/+ |
| 3c; Ext3b | *D. melanogaster* | ∆ppk23 |
| 3b,d; Ext3c,d | *D. yakuba* | ∆ppk23 |
| 3e; Ext3f; Ext4c | *D. yakuba* | w; UAS-GCaMP6s; ppk23-Gal4/+ |
| 3a,e | *D. melanogaster* | w; ppk23-Gal4; UAS.GCaMP6s |
| 3e; Ext4c | *D. yakuba* | ∆ppk23; UAS-GCaMP6s/+; ppk23-Gal4/+ |
| 3e; Ext4c | *D. yakuba* | w; UAS-GCaMP6s, ∆ppk25/∆ppk25; fru-Gal4/+ |
| 3f, Ext4b | *D. yakuba* | ∆ppk25 |
| 3g | *D. yakuba* | w; ppk23-Gal4/UAS-CsChrimson |
| 3g | *D. yakuba* | w; UAS-CsChrimson/+ |
| 4a, Ext5d,f,g | *D. yakuba* | w; 71G01-DBD/+; fru-p65.AD/UAS-CD8::GFP |
| 4b, Ext5e-g | *D. yakuba* | w; 71G01-DBD/+; dsx-p65.AD/UAS-CD8::GFP |
| 4a | *D. yakuba* | w; 71G01-DBD/UAS-GCaMP6s; fru-p65.AD/+ |
| 4b, 4f; Ext8e | *D. yakuba* | w; 71G01-DBD/UAS-GCaMP6s; dsx-p65.AD/+ |
| 4a, Ext7a | *D. yakuba* | w; 71G01-DBD/+; UAS-CsChrimson, fru-p65.AD/+ |
| 4b, Ext7b | *D. yakuba* | w; 71G01-DBD/+; UAS-CsChrimson, dsx-p65.AD/+ |
| 4c | *D. yakuba* | w; UAS-GCaMP6s/UAS-GCaMP6s; fru-Gal4/+ |
| 4d | *D. yakuba* | w; UAS-GCaMP6s/UAS-GCaMP6s; dsx-Gal4/+ |
| 4e | *D. yakuba* | w; ∆ppk25 UAS-GCaMP6s/∆ppk25 UAS-GCaMP6s; dsx-Gal4/+ |
| 4g | *D. yakuba* | w; 71G01-DBD/+; UAS-Kir2.1/+ |
| 4g | *D. yakuba* | w; 71G01-DBD/+; fru-p65.AD/UAS-Kir2.1 |
| 4g | *D. yakuba* | w; 71G01-DBD/+; dsx-p65.AD/UAS_Kir2.1 |
| Ext2a | *D. erecta* | UAS-CsChrimson::tdTomato /+; 71G01-Gal4/+ |
| Ext2b | *D. yakuba* | w; 71G01-AD/+; 15A01-DBD/UAS-CsChrimson |
| Ext3e; Ext10a | *D. melanogaster* | w; fru-Gal4/UAS-CD8::GFP |
| Ext3e; Ext10a | *D. yakuba* | w; fru-Gal4/UAS-CD8::GFP |
| Ext3e; Ext10b | *D. melanogaster* | w; dsx-Gal4/UAS-CD8::GFP |
| Ext3e; Ext10b | *D. yakuba* | w; dsx-Gal4/UAS-CD8::GFP |
| Ext3g | *D. yakuba* | ∆ppk23; UAS-GCaMP6s/+; dsx-Gal4/+ |
| Ext5b,f,g; Ext6a | *D. melanogaster* | w; 71G01-p65.AD/+; UAS-CD8::GFP, fru-DBD/+ |
| Ext5c,f,g; Ext6b | *D. melanogaster* | w; 71G01-p65.AD/UAS-CD8::GFP; dsx-DBD/+ |
| Ext6a | *D. melanogaster* | w; 71G01-p65.AD/+; UAS-GCaMP6s, fru-DBD/+ + |
| Ext6b | *D. melanogaster* | w; 71G01-p65.AD/UAS-GCaMP6s; dsx-DBD/+ |
| Ext6a; Ext7c | *D. melanogaster* | w; 71G01-p65.AD/+;UAS-CsChrimson, fru-DBD/+ + |
| Ext6b; Ext7d | *D. melanogaster* | w; 71G01-p65.AD/UAS-CsChrimson; dsx-DBD/+ |
| Ext10a | *D. melanogaster* | w; 15A01-p65.AD/+; 71G01-DBD/UAS-CD8::GFP |
| Ext10a | *D. yakuba* | w; 71G01-p65.AD/+; 15A01-DBD/UAS-CD8::GFP |
| Ext6c | *D. melanogaster* | w; UAS-GCaMP6s/+; dsx-Gal4/+ |
| Ext6c | *D. melanogaster* | w; UAS-GCaMP6s/+; dsx-Gal4/fru-Gal80 |
| Ext8c | *D. yakuba* | ∆OR67d |
| Ext10c | *D. melanogaster* | LexAop-SPA-T2A-SPA/+; fru-LexA/+ |
| Ext10c | *D. yakuba* | UAS-SPA-GFP/+; fru-Gal4/+ |

Supplementary Table 2. Female experimental genotypes by figure.

| Figure | Species | Female Genotype |
|---|---|---|
| 1b-d; 2b; 3a,b,d,e,f, 4a-e,g; Ext1a-c; Ext3c,d; Ext4b,c; Ext6a-c; ; Ext8a,d,e | *D. yakuba* | Wildtype (Ivory Coast) |
| 1b,c, 2b, 3a-c; 3e-g; 4a-e; Ext1a,c; Ext6a-c, ; Ext8a | *D. melanogaster* | Wildtype (Canton S) |
| 1b,c; 2b; 3e; 4a-e Ext1a,b,c; Ext6a-c | *D. simulans* | Wildtype |
| 1b,c; 2b; 3e; 4a-e; Ext1a; Ext6a-c | *D. erecta* | Wildtype (14021-0224.01) |
| 1b,c; Ext1a | *D. eugracilis* | Wildtype (SHL12) |
| 1b,c; Ext1a | *D. ananassae* | Wildtype (14024-0371.34) |
| Ext6a,b; Ext7c,d | *D. suzukii* | Wildtype (WT3) |
| 1d; 4f; Ext1d; Ext3f | *D. melanogaster* | PromE(800)-Gal4, tubP-Gal80$^{ts}$/UAS-StingerII, UAS-hid |

Supplementary Table 3. Detailed Statistics.

Supplemental Video 1. Courtship of a *D. melanogaster* female by a *D. melanogaster* male in the dark.

Supplemental Video 2. Courtship of a *D. simulans* female by a *D. simulans* male in the dark.

Supplemental Video 3. Courtship of a *D. yakuba* female by a *D. yakuba* male in the dark.

Supplemental Video 4. Optogenetic activation of Fru∩P1 neurons in a *D. yakuba* male toward a *D. melanogaster* female.

Supplemental Video 5. Optogenetic activation of Dsx∩P1 neurons in a *D. yakuba* male toward a *D. melanogaster* female.

Supplemental Video 6. Wing flicking behavior of *D. yakuba* male when approached by a *D. yakuba* male.
