# Supplementary material for: A modular circuit coordinates the diversification of courtship strategies
Source: Nature. 2024 Oct 9;635(8037):142–50. doi: 10.1038/s41586-024-08028-1 (PMC11540906; doi:10.1038/s41586-024-08028-1)
Supplement: Supplementary file 2 — Reporting Summary [file 41586_2024_8028_MOESM2_ESM.pdf]

Corresponding author(s): Vanessa Ruta
Last updated by author(s): Aug 16, 2024

Reporting Summary

Nature Portfolio wishes to improve the reproducibility of the work that we publish. This form provides structure for consistency and transparency in reporting. For further information on Nature Portfolio policies, see our Editorial Policies and the Editorial Policy Checklist.

Statistics

For all statistical analyses, confirm that the following items are present in the figure legend, table legend, main text, or Methods section.

n/a	Confirmed	
☐	☒	The exact sample size (*n*) for each experimental group/condition, given as a discrete number and unit of measurement
☐	☒	A statement on whether measurements were taken from distinct samples or whether the same sample was measured repeatedly
☐	☒	The statistical test(s) used AND whether they are one- or two-sided<br>*Only common tests should be described solely by name; describe more complex techniques in the Methods section.*
☐	☒	A description of all covariates tested
☐	☒	A description of any assumptions or corrections, such as tests of normality and adjustment for multiple comparisons
☐	☒	A full description of the statistical parameters including central tendency (e.g. means) or other basic estimates (e.g. regression coefficient) AND variation (e.g. standard deviation) or associated estimates of uncertainty (e.g. confidence intervals)
☐	☒	For null hypothesis testing, the test statistic (e.g. *F*, *t*, *r*) with confidence intervals, effect sizes, degrees of freedom and *P* value noted<br>*Give P values as exact values whenever suitable.*
☒	☐	For Bayesian analysis, information on the choice of priors and Markov chain Monte Carlo settings
☒	☐	For hierarchical and complex designs, identification of the appropriate level for tests and full reporting of outcomes
☐	☒	Estimates of effect sizes (e.g. Cohen's *d*, Pearson's *r*), indicating how they were calculated

Our web collection on statistics for biologists contains articles on many of the points above.

Software and code

Policy information about availability of computer code

Data collection	Behavioral data was collected using FlyTracker machine vision software (Caltech) and Flycapture2 Software(2.13.3.61) Development Kit (FLIR). Imagine data was recorded using PrairieView (5.5) software (Bruker). Fluorescence time-series were extracted using FIJI (2.14.0/1.54f, ImageJ, NIH).
Data analysis	Data was analyzed using Matlab 2022a, Prism 9, JAABA (0.5.0) machine learning sorftware (Janelia).

For manuscripts utilizing custom algorithms or software that are central to the research but not yet described in published literature, software must be made available to editors and reviewers. We strongly encourage code deposition in a community repository (e.g. GitHub). See the Nature Portfolio guidelines for submitting code & software for further information.

Data

Policy information about availability of data

All manuscripts must include a data availability statement. This statement should provide the following information, where applicable:

- Accession codes, unique identifiers, or web links for publicly available datasets
- A description of any restrictions on data availability
- For clinical datasets or third party data, please ensure that the statement adheres to our policy

Source data for all figures is available as supplementary material. All raw data underlying this study are available upon request from the corresponding author.

Research involving human participants, their data, or biological material

Policy information about studies with human participants or human data. See also policy information about sex, gender (identity/presentation), and sexual orientation and race, ethnicity and racism.

Reporting on sex and gender	n/a
Reporting on race, ethnicity, or other socially relevant groupings	n/a
Population characteristics	n/a
Recruitment	n/a
Ethics oversight	n/a

Note that full information on the approval of the study protocol must also be provided in the manuscript.

Field-specific reporting

Please select the one below that is the best fit for your research. If you are not sure, read the appropriate sections before making your selection.

☒ Life sciences ☐ Behavioural & social sciences ☐ Ecological, evolutionary & environmental sciences

For a reference copy of the document with all sections, see nature.com/documents/nr-reporting-summary-flat.pdf

Life sciences study design

All studies must disclose on these points even when the disclosure is negative.

Sample size	Preliminary experiments were used to assess variance. Sample sizes were not predetermined but kept consistent within comparison groups and comparable to other studies. Sample sizes are consistent with other recent and comparable studies: Sten et al, 2021, Chiu et al 2021, Roemschied et al, 2023.
Data exclusions	No data were excluded with one exception. For the D. yakuba P1 imaging, “splitP1” (71G01-AD 15A01-DBD intersection) had to be used rather than 71G01-Gal4 because this line was weak and bleached before an experiment could be completed. These splitP1 animals showed inter-animal variability in responses. Only ~1 in 5 (5/27) animals exhibited P1 responses to conspecifics, though in responding animals the responses were uniform and predictably evoked each time a male tapped a conspecific female (as shown in Fig. 2). We discovered this likely resulted from stochastic labeling of Fru- Dsx+ P1 subset. Specifically, splitP1 predominantly labels Fru+ P1 neurons, and labels 2-3 Fru- Dsx+ neurons in only some animals, as previously found in D. melanogaster57. Indeed, when we subsequently imaged Dsx∩P1 (71G01-DBD dsx-AD intersection) no inter-animal variability was observed and all animals responded to conspecifics with each tap. The same was found to be true when imaging for all Dsx+ projections in the LPC. Therefore, for simplicity and understanding we have presented have excluded the non-responsive D. yakuba splitP1 males in Fig. 2b.
Replication	All experiments were replicated using independent biological replicates over the course of days and typically across multiple months. All experiments were replicated across at minimum 4 animals. In cases where representative traces are depicted, they are shown alongside summary statistics for the entire dataset, typically 4-25 animals, depending on the experiment. All attempts at replication were successful.
Randomization	The order of female targets presented to males during imaging experiments was randomized each time an experiment was performed. Behavior experiments were loaded and analyzed by different experimenters. Comparison groups were interleaved on the same day in a order randomized by the loader and unknown to the analyzer until after analysis.
Blinding	All manually scored behavioral experiments were analyzed by an observer blind to the genotype, species, or treatment. The experimenter was not blind to male of female identities during functional imaging experiments.

Reporting for specific materials, systems and methods

We require information from authors about some types of materials, experimental systems and methods used in many studies. Here, indicate whether each material, system or method listed is relevant to your study. If you are not sure if a list item applies to your research, read the appropriate section before selecting a response.

Materials & experimental systems

n/a	Involved in the study	
☐	☒	Antibodies
☒	☐	Eukaryotic cell lines
☒	☐	Palaeontology and archaeology
☐	☒	Animals and other organisms
☒	☐	Clinical data
☒	☐	Dual use research of concern
☒	☐	Plants

Methods

n/a	Involved in the study	
☒	☐	ChIP-seq
☒	☐	Flow cytometry
☒	☐	MRI-based neuroimaging

Antibodies

Antibodies used	Primary antibodies: Chicken anti-GFP (Abcam ab13970), Mouse anti-brp (Developmental Studies Hybridoma Bank nc82), Rabbit anti-FruM (generated for this study by YenZyme against a synthesized peptide: HYAALDLQTPHKRNIETDV70), and Guinea pig anti-FruM, (gift from D. Yamamoto, Tohoku University).<br>Secondary Alexa Fluor antibodies were purchase from Life Technologies and used as dilution of 1/500. Secondary antibodies used were AF555 goat anti-Rat (A21434, Invitrogen), AF555 goat anti-Rabbit (A-21428, Invitrogen) AF647 goat anti- mouse (A-21235, Invitrogen), AF488 goat anti-chicken (A-11039, Invitrogen) and AF488 goat anti-rabbit (A11034, Invitrogen).
Validation	Chicken Polyclonal GFP antibody. Validated in WB, ICC/IF and tested in Tag - Aequorea victoria samples. Cited in 2531 publications.<br>Mouse anti-brp. Validated in IHC, IHC-IF, ICC, and IF. Cited in 1023 publications. Validation described in detail on the manufacturer's website.<br>For anti-FruM, this immunizing peptide was previously validate in Kimura et al, 2005 Nature.

Animals and other research organisms

Policy information about studies involving animals; ARRIVE guidelines recommended for reporting animal research, and Sex and Gender in Research

Laboratory animals	D. yakuba Wildtype (Ivory Coast)<br>D. melanogaster Wildtype (Canton S)<br>D. simulans Wildtype<br>D. erecta Wildtype (14021-0224.01)<br>D. eugracilis Wildtype (SHL12)<br>D. ananassae Wildtype (14024-0371.34)<br>All experimental animals were tested on days 4-7 post-eclosion.
Wild animals	This study does not involve wild animals.
Reporting on sex	The courtship behaviors described in this studied are sexually dimorphic and exclusive to male Drosophila
Field-collected samples	This study does not involve field-collected samples.
Ethics oversight	No ethical guidance or approval required.

Note that full information on the approval of the study protocol must also be provided in the manuscript.

Plants

Seed stocks	*Report on the source of all seed stocks or other plant material used. If applicable, state the seed stock centre and catalogue number. If plant specimens were collected from the field, describe the collection location, date and sampling procedures.*
Novel plant genotypes	*Describe the methods by which all novel plant genotypes were produced. This includes those generated by transgenic approaches, gene editing, chemical/radiation-based mutagenesis and hybridization. For transgenic lines, describe the transformation method, the number of independent lines analyzed and the generation upon which experiments were performed. For gene-edited lines, describe the editor used, the endogenous sequence targeted for editing, the targeting guide RNA sequence (if applicable) and how the editor was applied.*
Authentication	*Describe any authentication procedures for each seed stock used or novel genotype generated. Describe any experiments used to assess the effect of a mutation and, where applicable, how potential secondary effects (e.g. second site T-DNA insertions, mosiacism, off-target gene editing) were examined.*
